# The World’s Columbian Exposition

**Published:** 1892-09

**Authors:** 


### The World's Columbian Exposition.

But a few weeks now remain before the time of the dedication of the Exposition buildings, in October, yet the construction of the buildings is progressing so rapidly that we are encouraged to think all will be completed in time for that grand occasion. Invitations have been sent to many distinguished persons to attend these ceremonies; the invitation conveyed to the President of the United States has been accepted, and it is probable that every member of the President's Cabinet, and nearly every Senator, Congressman and Governor will be present. The music on that occasion will be specially grand. Sousa's Chicago Band will be a prominent participant; it is his intention to draw liberally on the best musical talent in this country and abroad in order that this new band may eclipse every other on this side of the Atlantic.

Antiquarians who expect to visit the World's Fair will have a treat in store for them; every department, almost, of the great Exposition will have its relics on view; those of Columbus alone will be very numerous; they will be brought from Spain, Italy, Rome, the West Indies, and other widely separated parts of the earth.

One of the best contributions from the States will be shown by Pennsylvania, the collection being furnished largely from Philadelphia under the auspices of a committee of its city council. Among the objects in this collection are the following: The chair occupied by Thomas Jefferson, when writing the Declaration of Independence; the table on which it was signed; the silver inkstand used on that occasion; Thomas Jefferson's sword; chair of memorial woods, including parts of Columbus' house in Spain; bell rung at Valley Forge when Washington occupied that place with his army; sofa belonging to George Washington; bench made from pew in old Christ Church occupied by Washington and Lafayette; punch bowl used by Washington and other revolutionary officers; first lightning rod invented by Ben. Franklin; electrical machine invented by Franklin; fans used by Franklin at the court of France when he was minister there; clocks of Benj. Franklin, William Penn and Oliver Crom well, running and keeping good time; Thomas Jefferson's thermometer; Pocahontas' necklace; surveying instrument used by William Penn in laying out the city of Philadelphia; the famous Liberty Bell, etc., etc.

In the Government building will be exhibited the original standard surveyor's chain, authorized by act of Congress, May 18, 1797, for executing surveys of Government lands. The chain was made by Benj. Rittenhouse, of Philadelphia, in 1797, and is still in the same hard wood box in which it was sent out by the manufacturer. B.

